# Mechanism of cognitive impairment after laparoscopic surgery and nursing application progress based on EEG monitoring

**DOI:** 10.3389/fneur.2025.1645814

**Published:** 2025-10-28

**Authors:** Juan Xu, Xuejiao Lv

**Affiliations:** ^1^General Surgery, Zhejiang Hospital, Hangzhou, China; ^2^Thoracic Surgery, Zhejiang Hospital, Hangzhou, China

**Keywords:** EEG monitoring, postoperative cognitive impairment, postoperative delirium, perioperative nursing, laparoscopy

## Abstract

As laparoscopic surgery becomes more complex, the early detection and management of postoperative neurological issues, particularly postoperative delirium and cognitive dysfunction, have gained prominence in clinical nursing. In recent years, electroencephalogram (EEG) monitoring has emerged as a non-invasive, real-time method for assessing brain function, and is increasingly being utilized in postoperative care. This includes evaluating the quality of anesthesia recovery, monitoring the balance of cerebral oxygen supply and demand, protecting neurological function, and overseeing postoperative sleep, demonstrating its potential benefits. This article provides a comprehensive review of the technical principles and application contexts of EEG monitoring in the nursing care of laparoscopic surgery patients, aiming to develop an optimized nursing model that encompasses the preoperative, intraoperative, and postoperative phases.

## 1 Introduction

Laparoscopic surgery is commonly utilized across various surgical specialties. As clinical demand rises, more intricate laparoscopic procedures that involve lengthy operation times, deep anatomical layers, significant technical challenges, and elevated perioperative risks are being performed. In the United States, 2 million cases of these surgeries are conducted each year (1). However, laparoscopic surgery continues to encounter technical challenges and potential complications. Most prior researches have concentrated on traditional complications like intraoperative bleeding and infections, while comprehensive studies on the objective monitoring and care of nerve damage and postoperative cognitive dysfunction—such as delirium and cognitive decline—are still limited. The underlying pathological and physiological mechanisms associated with laparoscopic surgery may significantly contribute to postoperative nerve damage and cognitive issues, with 10–50% of elderly patients experiencing delirium post-surgery (2). Postoperative delirium (POD) and postoperative neurocognitive dysfunction (POCD) are signs of reversible nerve injury, with incidence rates ranging from 36.6% in younger individuals to 42.4% in older adults (3), and they are closely linked to an increased risk of long-term dementia (4, 5). In the U.S., the healthcare costs related to POD are estimated at $32.9 billion annually (6), and the daily expenses for patients with delirium are double those of non-delirious patients, totaling between $38 billion and $152 billion each year (7).

Electroencephalography (EEG) serves as a non-invasive, cost-effective method for dynamically assessing fluctuations in brain function. It can provide a baseline evaluation of patients’ brain function prior to laparoscopic surgery, quantify anesthesia depth through EEG biomarkers during the procedure, and offer early warnings regarding cognitive recovery post-surgery, as well as indicate the extent of brain function recovery (8). For surgical nursing staff, enhancing perioperative management of patients under EEG monitoring is crucial for improving patient outcomes and alleviating the burden on families and society.

This review seeks to systematically combine EEG-based applications and creates an optimized nursing model aimed at minimizing neurological injury complications during laparoscopic surgery, particularly focusing on POD and POCD. The review will address the following scientific topics: (1) neurological complications following laparoscopic surgery; (2) the mechanisms behind POD and POCD; (3) how EEGs can help prevent and monitor these conditions; and (4) the development of a comprehensive postoperative care model for laparoscopic surgery utilizing EEG monitoring. By addressing these areas, the goal is to establish a monitoring system based on EEGs and develop an effective nursing approach that can reduce postoperative neurocognitive disorders, enhance patients’ quality of life, and alleviate the burden on families and the healthcare system.

## 2 Postoperative neurological complications after laparoscopic surgery

Postoperative neurological issues following laparoscopic surgery can arise from various factors, including pneumoperitoneum pressure, extended use of specific positions (like low head and high foot or lithotomy), and electrosurgical techniques. These complications may include autonomic dysfunction, peripheral nerve damage, and central nervous system issues. The likelihood of experiencing these complications is significantly influenced by the type of surgery performed, its duration, and the patient’s pre-existing health conditions.

### 2.1 Autonomic dysfunction

Once pneumoperitoneum is established, the rise in abdominal pressure affects the autonomic nervous system balance through several mechanisms: (1) Increased tension in the vagus nerve: Traction on the peritoneum activates the vagus nerve, which can result in bradycardia and, in severe instances, cardiac arrest (9). (2) Stimulation of the sympathetic nervous system: Elevated carbon dioxide levels (PaCO₂ > 50 mmHg) and the pressure from pneumoperitoneum activate the sympathetic nervous system, leading to significant fluctuations in blood pressure (over 20% from baseline) and postoperative nausea and vomiting (PONV) (10–13). The incidence of PONV can be as high as 80% in female patients, potentially due to hormonal changes and variations in drug metabolism (14).

### 2.2 Peripheral nerve complications

The primary causes of complications during surgical procedures are mechanical compression, electrical injury, and positional factors. Common types of nerve injuries include brachial plexus paralysis (15), obturator nerve injury (16), sciatic nerve injury (17), and femoral nerve injury (18), among others.

### 2.3 Central nervous system complications

Conventional complications of the central nervous system usually involve structural damage, like cerebral edema and cerebral infarction, as well as functional and metabolic disorders. These issues are uncommon in laparoscopic surgery and generally include brain injuries related to hypercapnia (19), cerebral infarction (20), and metabolic encephalopathy (21). Furthermore, POD and POCD are also considered central nervous system complications, but they are often categorized separately because of their distinct pathophysiological mechanisms and clinical presentations. This article primarily focuses on these two types of complications.

## 3 The manifestations and pathogenesis of postoperative neurocognitive disorders

### 3.1 Clinical manifestations and epidemiology

Postoperative neurocognitive dysfunction (PND) is a syndrome of cognitive impairment that occurs after surgery, caused by factors related to the surgery and anesthesia. It is a complex condition that primarily includes POD and POCD (22). These two conditions differ notably in their clinical symptoms, patterns of cognitive impairment, and epidemiological characteristics, as outlined below.

#### 3.1.1 POD

POD is defined as the sudden onset of impaired consciousness and cognitive dysfunction following surgery. It is marked by acute confusion, attention deficits, and fluctuating cognitive abilities. Symptoms include disorientation (misrecognition of time, place, and person), hallucinations or delusions, confused speech, behavioral agitation or suppression (“quiet delirium”), and temporary memory loss. The condition typically fluctuates significantly, usually emerging within 24 to 72 h after surgery and lasting from several hours to a few days. The overall incidence of POD is about 23%, but it can reach up to 50% in high-risk surgeries (23). In complex laparoscopic procedures, the incidence varies between 8 and 60%, depending on the surgical site and type (24–27). POD is closely linked to POCD, often considered an early stage of POCD, with an incidence ranging from 10 to 54% (28). The hallmark cognitive features of POD are acute impairments in attention and consciousness, affecting critical cognitive domains such as attention and awareness.

#### 3.1.2 POCD

POCD refers to ongoing cognitive decline following surgery, characterized by a notable reduction in abilities such as memory, attention, and executive function compared to pre-surgery levels. This impairment lasts for more than 1 week after the operation, often persisting for several weeks, months, or even longer (28, 29). Unlike the sudden and temporary changes seen in POD, POCD develops more gradually and is primarily evident through decreased ability to perform daily activities—for example, memory lapses causing repeated forgetting of medical instructions, and executive dysfunction leading to difficulties in completing complex tasks. The occurrence of POCD typically ranges from 10 to 54% and is closely linked to POD, with many studies suggesting that POD may be an early indicator of POCD (3, 26). Factors influencing its incidence include patient age, type of surgery, and preoperative cognitive reserve. Elderly patients and those undergoing complex laparoscopic procedures, such as radical gastrectomy, face a significantly higher risk of developing POCD (28, 29). This condition involves persistent impairment across multiple cognitive areas, particularly affecting memory, executive function, and the speed of information processing.

### 3.2 Mechanism

Postoperative neurocognitive impairment following laparoscopic surgery arises from various mechanisms, primarily neuroinflammation and disruptions in the cholinergic system. Additionally, certain complex laparoscopic procedures involve unique factors such as extended pneumoperitoneum and hypercapnia, which can lead to reduced blood flow to the brain and metabolic irregularities, further worsening the damage (Table 1).

**Table 1:** EEG correlation table of laparoscopic surgery specificity and anesthesia-related mechanisms.

Laparoscopic surgery-specific mechanisms	EEG
*Pneumoperitoneum-related injuries:* Pneumoperitoneum pressure (10-12 mmHg) leads to hypercapnia (PaCO₂ > 50 mmHg), causing cerebral vasodilation, increased intracranial pressure, and imbalance of cerebral oxygen supply and demand; At the same time, peritoneal traction activates the vagus nerve and exacerbates the neuroinflammatory response.	*α* inhibition: When the pneumoperitoneal pressure was > 10 mmHg, the frontal lobe *α* wave power decreased by > 30%, indicating that the brain perfusion was insufficient, and the pneumoperitoneum pressure and position needed to be adjusted together. *δ* dynamics: postoperative *δ* wave power increases by > 40% and lasts for > 6 h, which is associated with blood–brain barrier disruption and requires enhanced anti-inflammatory interventions (such as optimized fluid management); SEF: Intraoperative SEF ratio < 1.0 suggests inhibition of cerebral metabolism, and hypercapnia should be corrected in combination with blood gas analysis.
*Posture impact:* Special positions such as reverse Trendelenburg position change cerebral hemodynamics, resulting in cerebral hypoperfusion, prefrontal cortex and hippocampal metabolism abnormalities.	
*Surgical stress:* Prolonged surgery (>3 h) and electrosurgical procedures cause tissue damage, release pro-inflammatory factors such as IL-1, IL-6, TNF-*α*, disrupt the blood–brain barrier, activate microglia, and lead to a neuroinflammatory cascade.	
*Metabolic disorders:* Acidosis due to pneumoperitoneum affects synaptic function, mitochondrial electron transport chain abnormalities, decreased ATP production, and exacerbates neuronal damage.	
Anesthesia-related mechanisms	
*Direct drug inhibition:* Propofol, benzodiazepines inhibit the thalamic-cortical arousal system by enhancing GABA ergic neurotransmission, resulting in a decrease in *α* wave power and an increase in *δ* waves.	Abnormal BIS value; θ (4–8 Hz) are diffuse and can be partially reversed with discontinuation.
*Abnormal depth of anesthesia:* Excessive anesthesia at BIS<40 can lead to suppression of neuronal activity, while fluctuations in depth (BIS variant> 20) increase the metabolic burden on the brain.	
Cholinergic system interference: Anesthetic drugs reduce acetylcholine release, affect hippocampal memory function, and are directly related to postoperative cognitive decline.	

#### 3.2.1 Neuroinflammation and blood–brain barrier disruption

The involvement of neuroinflammation, disruption of the blood–brain barrier, and oxidative stress in postoperative neurocognitive disorders has been thoroughly researched and validated (30, 31). Studies (32) have shown that pro-inflammatory cytokines are increased in the serum and cerebrospinal fluid of POD and POCD. During surgical procedures, damaged tissues release oxygen free radicals, neurotrophic factors, interleukin-1, interleukin-6, tumor necrosis factor alpha (TNF-*α*), and other substances (33). These elements can either pass through the relatively permeable ventricles, actively cross the blood–brain barrier, or directly enter the brain through the compromised blood–brain barrier, where they bind to specific receptors in the central nervous system. This process activates microglia and endothelial cells, worsening postoperative inflammatory responses and leading to cognitive decline (34). Additionally, a study (35) using a rat model to investigate postoperative neurological dysfunction revealed issues with neurotrophic factors in memory-related regions like the hippocampus and prefrontal cortex. A recent prospective cohort study (36) indicated a direct link between POD and blood–brain barrier disruption, noting that the barrier’s permeability fluctuates with neuroinflammation and lactate levels, further influencing the onset of delirium. Furthermore, minimizing intraoperative bleeding may help reverse this disruption of the blood–brain barrier.

#### 3.2.2 Imbalance of cerebral oxygen supply and demand and perfusion abnormalities

Complex laparoscopic surgeries, particularly those involving the gastrointestinal tract, often necessitate a reverse Trendelenburg position and extended pneumoperitoneum to achieve an optimal surgical view and a smooth operating environment. Typically, the use of carbon dioxide pneumoperitoneum and the reverse Trendelenburg position can lead to significant alterations in cerebral blood flow and an increase in intracranial pressure (37, 38). Previous research (39) has shown that brain oxygenation notably declines when pneumoperitoneum pressure is maintained between 10 and 12 mmHg, which is linked to cognitive changes. A study examining the effects on short-term cognitive function in patients who underwent robot-assisted radical prostatectomy (40) revealed that some individuals experienced lower Mini Mental State Examination (MMSE) scores, POD, elevated intracranial pressure, and mild cognitive impairment. Furthermore, acidosis resulting from carbon dioxide pneumoperitoneum can disrupt synaptic nervous function, dilate cerebral blood vessels, raise intracranial pressure, enhance the release of vascular factors, and lead to venous short circuits, ultimately contributing to cognitive dysfunction (41).

#### 3.2.3 Oxidative stress and mitochondrial dysfunction

Mitochondria serve various roles, including metabolite and redox signaling, energy production in the form of adenosine triphosphate (ATP), and the regulation of nuclear gene expression and epigenetics. They respond to stress by continuously undergoing division and fusion (42, 43). Surgical stress can disrupt the mitochondrial electron transport chain. Recent studies (44) have indicated that disorders in mitochondrial energy metabolism and associated biological changes are closely linked to the development of neurocognitive disorders after surgery. The mechanisms behind the imbalance in mitochondrial homeostasis related to POCD involve mitochondrial dynamics and dysfunction (45). Furthermore, mitochondrial damage, such as oxidative stress, can cause an imbalance in the production and removal of oxygen free radicals, which can further impair mitochondrial function (43).

#### 3.2.4 Other factors such as surgical and anesthesia specificity

Complex laparoscopic surgeries involve high-risk procedures such as extended pneumoperitoneum, pelvic lymph node dissection (which can harm the autonomic nerve plexus), and gastrointestinal reconstruction (which may involve traction on the vagus nerve), all of which can have direct or indirect impacts on neurological function. Furthermore, the choice and dosage of anesthetic agents can influence the occurrence of postoperative neurocognitive disorders and elevate the risk of POD (46, 47). Recent research has also indicated that abnormal buildup of *β*-amyloid protein and synaptic function impairment (48, 49), along with disruptions in the gut-brain microbiota axis (50), can contribute to postoperative neurocognitive issues.

#### 3.2.5 Differences in mechanisms between POD and POCD

Although both POD and POCD fall under the category of PNDs and share fundamental pathological features like neuroinflammation and blood–brain barrier disruption, they differ significantly in terms of timing of injury, reversibility, and key mechanisms. These differences mainly highlight the contrast between “acute reversible injury” and “chronic potentially persistent injury”: (1) Timing of neuroinflammation: POD is closely linked to an acute neuroinflammatory response. Within hours to days following surgical trauma, pro-inflammatory factors (such as IL-6 and TNF-*α*) rapidly increase and penetrate the blood–brain barrier, triggering excessive microglial activation and a central acute inflammatory reaction. This inflammation typically diminishes gradually as systemic inflammation is controlled post-surgery and is characterized by short-term, intense effects (34, 36). In contrast, neuroinflammation in POCD presents as a subacute or chronic persistent condition. Although pro-inflammatory factor levels decrease after surgery compared to the acute phase seen in POD, the inflammation lasts much longer (up to several weeks or even months) and is accompanied by ongoing microglial activation and accumulation of neurotoxic substances, resulting in long-term damage to cognitive-related brain regions such as the hippocampus (30, 31). (2) Differences in the reversibility of blood–brain barrier damage: In patients with POD, blood–brain barrier disruption is dynamically reversible and is mainly influenced by factors like acute hypercapnia and fluctuations in intracranial pressure during surgery. Research indicates that minimizing intraoperative bleeding can significantly reverse this abnormal permeability (36). As pneumoperitoneum is relieved and the balance between cerebral oxygen supply and demand is restored, blood–brain barrier function can gradually recover over several days. In contrast, blood–brain barrier damage in POCD tends to cause structural alterations. Prolonged inflammatory stimulation leads to ongoing disruption of tight junctions in vascular endothelial cells, along with the accumulation of toxic substances such as beta-amyloid, making full restoration of barrier function difficult and resulting in axonal injury and synaptic loss (49). (3) Differences in the duration of cerebral metabolism and perfusion abnormalities: The imbalance between cerebral oxygen supply and demand in POD primarily arises from acute intraoperative events, such as sudden increases in pneumoperitoneum pressure (notably reducing cerebral oxygenation at 10–12 mmHg) and transient cerebral blood flow fluctuations caused by specific surgical positions. These issues can be rapidly resolved by adjusting pneumoperitoneum pressure and optimizing patient positioning, making the metabolic disturbances mostly temporary (39, 40). Conversely, POCD is linked to prolonged cerebral hypoperfusion and reduced metabolic reserve. Even if oxygenation is restored during surgery, cognitive-related brain regions (like the prefrontal cortex) may remain in a low metabolic state for an extended period due to persistent mitochondrial dysfunction (including insufficient ATP production and buildup of oxidative stress products) and impaired cerebral blood vessel autoregulation. (4) Differences between the cholinergic system and synaptic damage: In POD, inhibition of the cholinergic system is an acute functional suppression, primarily due to short-term acetylcholine depletion caused by anesthetic agents (like anticholinergic drugs) and surgical stress. This results in sudden symptoms such as confusion and attention deficits. Once the medication is stopped or stress is alleviated, normal function can quickly return (41). In contrast, POCD involves structural damage to cholinergic neurons, with prolonged inflammation and oxidative stress causing cell death and reduced synaptic density in the basal forebrain. It is also linked to impaired synaptic plasticity (for example, dysfunction of neurotrophic factors), leading to lasting impairments in cognitive areas like memory and executive function, with longer recovery times and a risk of progressing to chronic cognitive decline (34, 48). (5) Other specific mechanistic differences: POD is also associated with acute disruptions in the sleep–wake cycle and temporary functional disconnection of the default mode network (DMN). EEG findings may show sudden increases in *δ* power and a shift toward lower frequencies in the dominant rhythm (51, 52). On the other hand, POCD is more related to chronic pathological processes such as long-term disturbances in the gut microbiota-brain axis and abnormal *β*-amyloid protein accumulation. These factors continuously impact neuroendocrine and immune regulation pathways, causing gradual cognitive decline (49, 50). In summary, POD is characterized by acute, reversible, and functional impairments, whereas POCD involves chronic, potentially lasting, and structural damage. Understanding these mechanistic differences offers a theoretical foundation for accurate diagnosis and targeted treatment, including clinical approaches like EEG monitoring.

## 4 Diagnosis of POD and POCD

The primary clinical assessment tools for diagnosing POD include the Confusion Assessment Method (CAM) (53), the ICU Confusion Assessment Method (CAM-ICU), and additional tools like the Delirium Rating Scale and the 4AT Scale (54). CAM is considered the gold standard for assessing POD (55). Diagnosing postoperative cognitive dysfunction primarily depends on neuropsychological tests such as the MMSE and the Montreal Cognitive Assessment (MoCA) (56). Due to technical challenges and complex procedures, there are limited clinical applications for biomarker detection, including cerebrospinal fluid analysis and blood markers related to astrocyte injury and blood–brain barrier disruption, as well as functional magnetic resonance imaging to monitor abnormalities in hippocampal blood oxygen levels and PET-CT for brain metabolism assessment (56). However, it is undeniable that some postoperative patients, particularly elderly individuals, show low cooperation with scale assessments, and these assessment methods are often affected by the experience and subjectivity of medical personnel. International studies have found that nurses’ sensitivity and specificity in evaluating delirium were 47 and 98%, respectively (57). This finding is not isolated; several international multicenter studies have confirmed that nursing staff commonly exhibit insufficient sensitivity when using traditional delirium assessment tools. For instance, a study involving 234 elderly hospitalized patients across 12 countries (55) found that despite standardized training, nurses using the CAM scale to detect delirium had an overall sensitivity of only 51%. This was mainly due to the fluctuating nature of delirium (such as “quiet delirium” being easily missed) and variations in nurses’ ability to recognize subtle symptoms. Additionally, a meta-analysis focusing on ICU patients (57) reported that the sensitivity of nursing staff using only the CAM-ICU ranged from 35 to 58%, which was significantly lower than the 82 to 91% sensitivity achieved through assessments by multidisciplinary teams, highlighting the limitations of traditional tools in nursing practice.

In recent years, optimization tools for nursing situations have increasingly been implemented in clinical practice, partially addressing the limitations of traditional methods: (1) 3-min Diagnostic Interview for Delirium (3D-CAM): This simplified version of the CAM tool targets key symptoms such as “acute onset, attention deficits, and altered consciousness” through structured questions. A recent systematic review and meta-analysis (58) reported that 3D-CAM has a sensitivity of 92% and specificity of 95%, with an administration time reduced to under 3 min, making it well-suited for busy postoperative nursing environments. (2) Nursing Delirium Screening Scale (Nu-DESC): This bedside screening tool is specifically designed for nursing staff. By evaluating five symptoms—restlessness, inattention, clouded consciousness, hallucinations, and fluctuation—its sensitivity ranges from 76 to 82% in elderly postoperative patients, and its agreement with neurologists’ diagnoses is significantly better than that of CAM-ICU (55). Nonetheless, CAM/CAM-ICU remains the predominant tool in clinical use, and the adoption and standardization of alternative tools require improvement. Therefore, it is essential to accurately detect postoperative neurocognitive disorders through technological advancements and the development of an objective evaluation system based on EEG monitoring. EEG can provide an objective complement to scale assessments, creating a combined “subjective + objective” evaluation model alongside scales, thereby enhancing the early detection accuracy of delirium. This approach not only impacts patient outcomes but also reduces healthcare costs, representing a crucial strategy to address the challenges posed by an aging population (Table 2).

**Table 2:** Comparison of POD and POCD.

Identification point	POD	POCD
Clinical manifestations	Acute confusion; Disorientation, hallucinations, fluctuating between wakefulness and drowsiness	Memory loss; Difficulty concentrating, executive dysfunction, and no obvious impairment of consciousness
Time of onset	24–72 h after surgery; Lasts from several days to 1 week	1–3 months after surgery; Last from several months to several years. At least 10% of patients over 60 years of age develop persistent POCD 3 months after surgery (66).
EEG features		
Preoperatively	Frontal lobe high α power; α function connection enhancement; Lower and preoperative *γ* bands are lower	The lower α frequency band has higher power and lower α peak frequency
During the operation	BIS < 40	
Postoperatively	*α* frequency band power decreases; *δ* frequency band power increases	
	*δ* frequency band power increases; α frequency band recovery delay; DMN connection strength decreases; PDR becomes low frequency (<8 Hz).	Power peaks appeared in the θ band; α band successively and persisted
Assessment tools	CAM, CAM-ICU, 3D-CAM, Nu-DESC, delirium rating scale and 4AT scale focus on state of consciousness assessment	MMSE, MoCA, with a focus on cognitive domain testing
Focus on care	Real-time monitoring of delirium warnings (*δ*/*α* > 1.5) to prevent accidental injuries and regulate sleep–wake cycles	Long-term cognitive rehabilitation to improve α rhythm and enhance DMN connectivity (e.g., cognitive training, music therapy)
Prognostic association	Associated with increased short-term mortality, with a reversible rate of about 70%	Associated with an increased risk of long-term dementia, approximately 30% develop chronic cognitive impairment

## 5 The role and nursing application of EEG in preventing and monitoring postoperative cognitive impairment

EEG monitoring is a non-invasive, real-time, and cost-effective method for assessing brain function, proving to be particularly valuable in systemic neuroprotective strategies related to POD and POCD (59). Research indicates (60–62) that POD and POCD are associated with specific spectral features of EEG, particularly frontal lobe alpha waves, which reflect the balance between arousal and sedation. This balance can be disrupted by neuroinflammation and anesthesia responses during surgery. The American Society for the Advancement of Rehabilitation and Perioperative Quality advises anesthesiologists to utilize EEG, including raw waveforms, spectrograms, and processed indices, to guide anesthesia (59). The characteristics of preoperative raw EEG, such as time-frequency and power spectrum analysis, along with the dual frequency index (BIS) derived from complex EEG algorithms, may serve as predictive markers for POD and POCD in patients (63, 64). It is important to note that the BIS primarily indicates the depth of anesthesia, and its link to POD/POCD is largely based on pharmacological factors like excessive sedation from anesthetic drugs and individual patient sensitivity. However, physiological stressors unique to laparoscopic surgery—such as pneumoperitoneum pressure, CO₂ retention, and changes in cerebral perfusion—can cause neurocognitive impairment through mechanisms unrelated to anesthesia depth, including neuroinflammation and imbalances in cerebral oxygen supply and demand (Table 1). For instance, (1) certain positions (like Trendelenburg) can reduce venous return, resulting in inadequate cerebral perfusion; (2) hypercapnia from carbon dioxide pneumoperitoneum can diffuse into brain tissue, lower cerebrospinal fluid pH, inhibit brain cell metabolism, cause cerebral vasodilation, increase cerebral blood flow, and raise intracranial pressure, all of which affect neuronal electrical activity (65). These pathological and physiological changes are directly observable in the EEG, making EEG a vital tool for monitoring brain function during the perioperative period. Common EEG indicators include: suppressed *α* power, which signals reduced attention or metabolic inhibition; increased *δ* power, linked to postoperative delirium and cerebral metabolic disturbances; and abnormal *γ* frequency bands, which suggest neuroinflammation or synaptic dysfunction. By continuously monitoring these biomarkers, clinical teams can identify patients at high risk early on and adjust perioperative management accordingly—such as optimizing ventilation settings, controlling pneumoperitoneum pressure, or applying neuroprotective measures—to help lower the incidence of POD/POCD.

Perioperative care is essential for ensuring patient safety and facilitating recovery, encompassing everything from pre-surgery preparation to long-term follow-up. In the context of high-risk postoperative care, like laparoscopic surgery, nursing teams can greatly minimize complications, speed up recovery, and enhance patients’ quality of life by employing comprehensive interventions and collaborating across disciplines, all while utilizing accurate and objective EEG monitoring.

### 5.1 Preoperative evaluation and cognitive rehabilitation intervention

Identifying patients at risk for POD and POCD after laparoscopic surgery early on can facilitate the implementation of timely brain health interventions and optimal care strategies, preventing irreversible brain function deterioration. Analyzing multi-lead raw EEG data from perioperative patients can yield valuable cognitive-related insights. The alpha frequency band of EEG, which originates from the thalamus, plays a role in regulating wakefulness, attention, and other essential cognitive functions. Previous research (51) has indicated that preoperative high alpha wave power, increased alpha functional connectivity, and structural damage in the frontal lobe can help identify patients at high risk for POD. In elderly patients undergoing abdominal surgery, those with preoperative cognitive decline show reduced alpha frequency power and peak alpha frequency during anesthesia maintenance, possibly because these patients are more likely to reach deep anesthesia with standard anesthesia doses (67). This finding aligns with a study that examined preoperative biomarkers linked to delayed neurocognitive recovery (68), which found that patients experiencing delayed recovery after surgery exhibited higher power in the low alpha frequency band of baseline EEG and lower alpha peak frequency.

Besides the alpha frequency band, other EEG frequency band indicators are also linked to postoperative cognitive dysfunction. A separate study (69) investigating the connection between changes in perioperative EEG and POD in older patients revealed that the preoperative low spectral edge frequency (SEF) and gamma band (30.1-45 Hz) in those who experienced POD were significantly lower compared to those who did not. An SEF of 17.75 Hz or lower before surgery showed high sensitivity (94.4%) and a negative predictive value (97.7%) for POD. The SEF ratio for patients with POD from wakefulness to anesthesia induction was nearly 1, while for those without POD, it was above 1, suggesting that POD patients did not exhibit the typical EEG slowing response. Thus, preoperative SEF, SEF ratio, and gamma band power can serve as independent predictors of POD.

The primary factors that can interfere with EEG monitoring include physiological disturbances, equipment and external influences, effects of anesthesia and medications, as well as operational and technical issues. To enhance the accuracy of EEG monitoring, thorough preoperative preparation and adherence to standardized procedures are essential. This includes: (1) Skin preparation: cleaning the skin with alcohol wipes and scrubs to minimize skin impedance (target ≤ 5KΩ) and applying conductive paste to improve electrode contact and decrease electromyographic noise; (2) Proper electrode placement: utilizing the international 10–20 system for positioning, particularly focusing on the frontal, temporal, and parietal leads.

EEG monitoring in preoperative assessments indicates that alpha attenuation during periods of wakefulness, rest, and eye opening is linked to postoperative attention issues (70). Consequently, when baseline EEG data is available, nursing staff can identify high-risk patients for cognitive impairment using specific EEG biomarkers and tools like the MMSE and 4AT scales. Researches (29, 71) have identified age, preoperative cognitive deficits, stroke, and other health conditions as risk factors for postoperative neurological issues. While identifying high-risk patients with cognitive impairments does not alter the surgical method, it allows for the creation of a tailored plan with the anesthesiology team prior to surgery, focusing on manageable factors such as intraoperative hypotension, cerebral perfusion, and anesthesia duration and depth to mitigate the risk of POD and POCD. Additionally, a recent study (72) involving 251 elderly patients undergoing major non-cardiac surgery found that those who participated in 10 days of cognitive training or 1 hour of cognitive exercises before surgery experienced a reduction in POD rates from 23.0 to 14.4%, although this difference was not statistically significant (*p* = 0.08). This suggests a potential avenue for future research, indicating that cognitive pre-habilitation may offer advantages for high-risk patients, warranting further trials to confirm its effectiveness.

### 5.2 Intraoperative monitoring and anesthesia depth optimization intervention

In 1937, Gibbs et al. were the first to observe the impact of anesthetics on EEG, leading to the idea of using EEG to monitor the depth of anesthesia. Since the 1990s, EEG has become a common tool for assessing anesthesia and sedation levels in clinical settings (73). The European Society of Anesthesiology now recommends EEG-guided anesthesia monitoring to help prevent POD and POCD (74). Currently, quantitative EEG indicators are primarily utilized to monitor anesthesia depth during surgery, aiming to prevent intraoperative awareness or excessive anesthesia, thus minimizing perioperative complications, particularly postoperative neurocognitive disorders. Commonly used clinical indicators include the Bispectral Index (BIS), Patient State Index (PSI), entropy index, and phase lag entropy (PLE) (75, 76). Among these, BIS is the most frequently used EEG monitoring tool in clinical practice, with its values closely linked to consciousness levels. Typically, loss of consciousness is observed at BIS values between 68 and 75 (77), while values between 40 and 60 indicate adequate maintenance of general anesthesia during procedures (78). Monitoring BIS intraoperatively helps prevent excessive anesthesia and significant fluctuations in anesthesia depth, thereby reducing cognitive impairment associated with surgery and anesthesia. A randomized controlled study (79) involving non-cardiac and non-neurological surgeries found that patients over 75 who experienced POD had lower BIS values compared to those without delirium. A predictive model incorporating BIS, MMSE, anemia, daily living activities, and blood urea nitrogen could serve as a tool for forecasting POD in older patients. In a trial of non-cardiac surgeries (80), including laparoscopic procedures, it was observed that patients who had BIS-guided anesthesia and maintained BIS values between 40 and 60 experienced a lower rate of POD and POCD with appropriate care. Another prospective controlled study (81) using attention network testing indicated that the BIS group significantly reduced the total dosage of propofol and remifentanil compared to the non-BIS group. BIS monitoring during anesthesia can facilitate quicker cognitive recovery and decrease acute delirium in elderly patients undergoing colon cancer surgery. A meta-analysis of clinical studies (82) conducted in 2020 also supported the conclusion that BIS monitoring has a protective effect against POD on day 1 and POCD on day 30. Furthermore, combining near-infrared spectroscopy with EEG can help identify intraoperative cerebral hypoperfusion events, particularly in situations where pneumoperitoneum raises intracranial pressure during complex laparoscopic surgeries.

It is important to note that anesthetics are typically identified by their effects on BIS values and a widespread increase in *θ* waves, whereas the physiological stress from laparoscopic surgery primarily appears in the EEG as a reduction in alpha waves (<8 Hz) and an increase in *δ* power (1–4 Hz), which coincide with a decrease in cerebral oxygen saturation.

### 5.3 Postoperative monitoring and neurocognitive rehabilitation intervention

The occurrence of POD and POCD is linked to the inhibition of the central cholinergic system and reduced neuronal activity. Research indicates that a decrease in alpha frequency band power (8–13 Hz) and an increase in delta frequency band power (1–4 Hz) following surgery may indicate a disruption in the thalamocortical feedback mechanism and mitochondrial synaptic dysfunction (83). This serves as the pathophysiological foundation for utilizing postoperative EEG to monitor neurocognitive disorders in patients. The systemic inflammatory response triggered by surgical trauma intensifies neuroinflammation via the blood–brain barrier, which inhibits brain electrical activity and disrupts cognitive network connectivity. An increase in slow wave activity observed in postoperative EEG may signify a mismatch between the brain’s metabolic demands and oxygen supply, directly correlating with the onset of delirium. Consequently, the rise in delta frequency band power and the delayed recovery of alpha frequency band activity within 24–72 h post-surgery are associated with delirium occurrence (84). Additionally, integrating the CAM-ICU scale can enhance diagnostic accuracy (85). In the postoperative phase, one-minute EEG monitoring from a single channel (Fp2-Pz) revealed a significant increase in delta frequency band power among elderly patients with PND (52). When POD manifested, the posterior dominant rhythm (PDR) was found in the lower frequency band, and the severity of delirium was positively correlated with the relative power of the occipital lobe theta waves. Over time, patients with cognitive impairment showed a shift in EEG relative power toward higher frequencies, with power spectral peaks emerging in the theta and alpha bands, indicating cognitive recovery (60). Furthermore, studies have shown that POD is linked to a reduction in the connectivity strength of the default mode network (DMN) as observed in EEG, with surgical stress and inflammation causing functional disconnection within the DMN, leading to decreased correlation between the posterior cingulate gyrus and the frontal lobe (86). Evidence supporting the use of quantitative EEG monitoring for POD, based on increased relative delta power and decreased beta power, is growing (87). Nursing staff can help mitigate patients’ exposure to blue light at night, enhance natural daylight, and lower the risk of POD by regulating alpha wave rhythms, as research suggests that lighting can influence the power of certain EEG waves, particularly alpha waves (88). For patients at high risk for postoperative neurocognitive disorders, multimodal interventions such as EEG neurofeedback training to boost alpha waves (89), music therapy to synchronize theta/gamma waves (90), and cognitive training to enhance DMN connectivity (91) can be employed to facilitate cognitive rehabilitation.

EEG is capable of not only tracking the typical changes associated with POD and POCD but also offering early indications of potential neurocognitive disorders by assessing sleep quality. Researches indicate (92, 93) that poor sleep quality is linked to a higher risk of POD, and EEG monitoring provides a more objective measure of patients’ sleep quality. EEG serves as a crucial diagnostic tool for sleep disorders, and when used alongside polysomnography, it allows for a thorough evaluation of sleep structure and irregular patterns. For instance, patients with insomnia often experience delayed sleep onset, reduced N3 sleep phase, and the presence of high-frequency beta waves (94). When detrimental changes in brainwaves affecting sleep are identified, this can provide objective evidence for nursing staff. Numerous large-scale meta-analyses (95, 96) have demonstrated that using earplugs, either alone or in combination with eye masks, can enhance sleep quality and help prevent delirium in postoperative patients. Furthermore, a clinical study (97) on the effectiveness of biofeedback therapy for insomnia revealed that a biofeedback approach, which modifies EEG and electromyography power, can enable patients to gain voluntary control over their physiological timing, thus alleviating sleep disorders and indirectly lowering the risk of POD.

The use of EEG monitoring, guidance, and treatment during sleep offers nursing staff a direct and non-invasive approach to enhance patient sleep quality, particularly in noisy settings like the ICU. In the future, advancements in portable EEG monitoring devices and further research on perioperative EEG characteristics may enable clinical nursing staff to establish warning thresholds for POD by continuously monitoring and analyzing EEG data. For patients at high risk of postoperative cognitive decline, early cognitive rehabilitation training can be initiated.

EEG monitoring can help eliminate evaluation bias, whether it is used preoperatively, intraoperatively, or postoperatively, especially for patients who are comatose or have language impairments. Additionally, it allows for real-time monitoring of brain function changes, facilitating personalized interventions. Its objectivity, real-time capabilities, and non-invasive nature are leading to its increasing adoption throughout the entire surgical process (Table 3).

**Table 3:** Framework of perioperative EEG monitoring optimized nursing mode.

Section	Preoperative	Intraoperative	Postoperative
EEG markers	Frontal α power; SEF; γ frequency band power	BIS; Brain oxygen saturation (near-infrared spectroscopy combined with EEG)	δ and α band recovery delay; Sleep architecture; Weakened DMN connectivity
Potential mechanisms	Baseline cognitive impairment	Cerebral hypoperfusion/CO₂ retention	Neuroinflammation / Metabolic Suppression
Nursing intervention			
Time point	Admission assessment and pre anesthesia visit	Anesthesia induction until the end of the surgery	Before discharge
Core monitoring	EEG; MMSE/4AT	BIS monitoring; Dynamic SEF tracking; Cerebral oxygen saturation measurement	EEG; Sleep architecture analysis; Daily CAM-ICU assessment;
Intervention strategy	Identifying high-risk patients; Optimizing anesthesia plans; Cognitive pre rehabilitation	Adjust the pneumoperitoneum pressure and appropriately adjust the patient’s position to maintain normal PaCO₂ levels; Regulate the depth of anesthesia	Reduce blue light exposure, music therapy; Sleep optimization (earplugs, eye masks); Cognitive rehabilitation (multimodal cognitive stimulation)
Expectations	Identify high-risk populations for POD/POCD; Reduce the risk of intraoperative brain injury	Avoid excessive or insufficient anesthesia to maintain the balance between brain metabolism and supply–demand	Early identification of delirium; Promotion of cognitive function recovery; Reduction of long-term risks

## 6 Benefits

By enhancing perioperative management strategies, such as implementing an optimized nursing model based on EEG monitoring, numerous advantages can be realized across various levels. For patients, this approach can decrease POD, alleviate pain and complications, speed up recovery, lessen the caregiving burden on families by supporting independent living, and effectively prevent chronic cognitive decline and long-term mortality. For healthcare professionals, it can enhance their skills while minimizing medical disputes and reducing occupational stress. On a societal level, this intervention model can lead to significant reductions in acute hospitalization costs and long-term care expenses, improve the allocation of medical resources, and provide public health benefits by easing the burden on families and society, as well as facilitating the recovery of work capabilities. In the long term, it will also encourage advancements in anesthesia monitoring technology and the development of the smart healthcare industry, fostering a positive cycle of policy support and coordinated industry growth.

POD and POCD following laparoscopic surgery arise from a combination of various high-risk factors, including older age, pre-existing cognitive issues, and the toxicity of anesthesia drugs, as well as pathological processes like neuroinflammation and disruptions to the blood–brain barrier. In current clinical practice, while some preoperative risk assessment methods (such as the Mini Mental State Examination, tailored anesthesia plans, and cognitive pre-habilitation) have been implemented in perioperative care, their effectiveness is hindered by challenges like the absence of real-time neurological monitoring tools, reliance on subjective assessment scales, and difficulties in detecting subtle brain function impairments during surgery. Additionally, the relationship between cognitive pre-habilitation before surgery and anesthesia management during the procedure remains unclear, and there is a lack of targeted interventions for postoperative sleep disorders based on EEG data. Therefore, a comprehensive strategy that includes accurately identifying high-risk patients preoperatively (for instance, by combining EEG alpha wave power with inflammatory marker levels), optimizing anesthesia depth dynamically during surgery, and providing early warnings and interventions postoperatively (such as monitoring EEG slow wave activity related to sleep deprivation) could help reduce the rates of POD and POCD. EEG monitoring offers a promising approach for “early warning and early intervention” in addressing POD and POCD by tracking changes in brain function, thereby lowering the risk of neurological complications. Consequently, a “preoperative, intraoperative, postoperative” precision nursing model is proposed. Future efforts should focus on developing portable EEG devices and deep learning-based EEG pattern recognition systems to assess the effectiveness of preoperative cognitive pre-habilitation, enable real-time alerts for postoperative neurocognitive dysfunction, and incorporate these advancements into standardized nursing protocols, ultimately achieving precision nursing objectives. With advancements in this research, EEG monitoring could evolve from being merely an “auxiliary tool” to a “decision engine,” enhancing postoperative neurocognitive care from an empirical to a precision medicine approach.
